# The higher the household income, the lower the possibility of depression and anxiety disorder: evidence from a bidirectional Mendelian randomization study

**DOI:** 10.3389/fpsyt.2023.1264174

**Published:** 2023-11-20

**Authors:** Guangyan Liu, Wenlin Liu, Xifeng Zheng, Junyan Li

**Affiliations:** ^1^Department of Geriatrics, Affiliated Hospital of Guangdong Medical University, Zhanjiang, China; ^2^Department of Traditional Chinese Medicine, Affiliated Hospital of Guangdong Medical University, Zhanjiang, China; ^3^Department of Cardiology, Affiliated Hospital of Guangdong Medical University, Zhanjiang, China

**Keywords:** household income status, causal relationship, Mendelian randomization study, depression, anxiety disorder

## Abstract

**Objectives:**

Observational studies have demonstrated that household income is associated with morbidity of mental disorders. However, a causal relationship between the two factors remains unclear. Therefore, we investigated the causal relationship between household income status and genetic liability of mental disorders using a bidirectional Mendelian randomization (MR) study.

**Methods:**

This MR study included a large cohort of the European population from publicly available genome-wide association study datasets. A random-effects inverse-variance weighting model was used as the main standard, with MR-Egger regression, weighted median, and maximum likelihood estimations performed concurrently as supplements. Sensitivity analysis, consisting of heterogeneity and horizontal pleiotropy tests, was performed using Cochran’s Q test, MR-Egger intercept, and MR-PRESSO tests to ensure the reliability of the conclusions.

**Results:**

A higher household income tended to be associated with a lower risk of genetic liability for depression (odds ratio [OR]: 0.655, 95% confidence interval [CI] = 0.522–0.822, *p* < 0.001) and anxiety disorder (OR: 0.666, 95% CI = 0.526–0.843, *p* < 0.001). No associations were observed for schizophrenia (OR: 0.678, 95% CI = 0.460–1.000, *p* = 0.05), panic disorder (OR: 0.837, 95% CI = 0.445–1.577, *p* = 0.583), insomnia (OR: 1.051, 95% CI = 0.556–1.986, *p* = 0.877), obsessive-compulsive disorder (OR: 1.421, 95% CI = 0.778–2.596, *p* = 0.252), and bipolar disorder (OR: 1.126, 95% CI = 0.757–1.677, *p* = 0.556). A reverse MR study showed no reverse causal relationship between psychiatric disorders and household income. Sensitivity analysis verified the reliability of the results.

**Conclusion:**

Our results revealed that the population with a higher household income tended to have a minor risk of genetic liability in depression and anxiety disorders.

## 1 Introduction

Mental disorders encompass a broad spectrum of illnesses, including schizophrenia, anxiety disorders, depressive disorders, and bipolar disorder, and represent a significant public health concern. These disorders are characterized by a combination of abnormal thoughts, perceptions, emotions, behaviors, and interpersonal relationships. According to 155 epidemiological investigations conducted in 59 countries, approximately 17.6% of adults experience a common mental disorder within a 12-month period. Moreover, the aggregated lifetime prevalence estimates from 85 surveys across 39 countries suggest that around 29.2% of individuals have experienced a common mental disorder at some point in their lives (1). Furthermore, mental disorders rank among the top 10 leading causes of global burden (2). The profound impact of these disorders on individuals, families, and societies cannot be overstated.

In the context of the global economic recession, the potential association between declining household income and mental disorders has gained increasing attention. Several observational studies have reported a correlation between household income and mental disorders (3–5). However, such observational studies come with inherent limitations such as a lack of randomization, measurement errors, challenges in controlling variables, and potential for bias (6). To date, limited evidence exists regarding the causal relationship between household income status and mental disorders, particularly owing to the scarcity of large-sample cohort studies. Considering the above, further research is needed to unravel the intricate relationship between household income and mental disorders, facilitating a comprehensive understanding of how household income impacts mental health outcomes. This knowledge will pave the way for the development of evidence-based interventions and policies aimed at mitigating the effects of household income disparities on mental health.

Mendelian randomization (MR) is a statistical method extensively employed in epidemiology and genetics studies to discern causal relationships between exposure factors and outcomes (7, 8). MR is based on Mendel’s law of inheritance, which describes how genetic variants are allocated randomly during meiosis (9). MR utilizes instrumental variables, notably genetic variations such as single nucleotide polymorphisms (SNPs) linked to a risk factor of concern (e.g., household income status), to explore whether the chosen risk factor has a causal impact on the outcome of interest (e.g., mental disorders) (10). In the absence of randomized controlled trials (RCTs), MR studies serve as an alternative strategy for causal inference because genetic variants are subject to random assignment during meiosis mirroring the RCT process. Consequently, MR has advantages over traditional observational studies, minimizing confounding risks and elucidating reverse causality, rendering it a powerful instrument for exploring causality in epidemiological research (11). Furthermore, MR studies have demonstrated their efficacy in probing the causal connections among behavioral exposure, educational attainment, household income circumstances, and a broad spectrum of diseases (12–14).

The primary objective of this study was to investigate the bidirectional causal relationship between genetic predisposition associated with household income status and the occurrence of common mental disorders. This investigation was conducted utilizing an MR approach, leveraging a substantial cohort of individuals from the European population as sourced from publicly accessible genome-wide association study (GWAS) datasets.

## 2 Materials and methods

### 2.1 Fundamental assumptions of MR study and MR study design

To achieve impartial results, an MR study depends on three fundamental assumptions: (1) Relevance—the selected genetic instrumental variables (IVs) are significantly associated with the exposure factor; (2) Independence—the IVs are independent of potential confounders associated with exposure factors and outcomes; and (3) Exclusion restriction—the IVs affect the outcomes only through the exposure factor (15).

**Figure 1:**
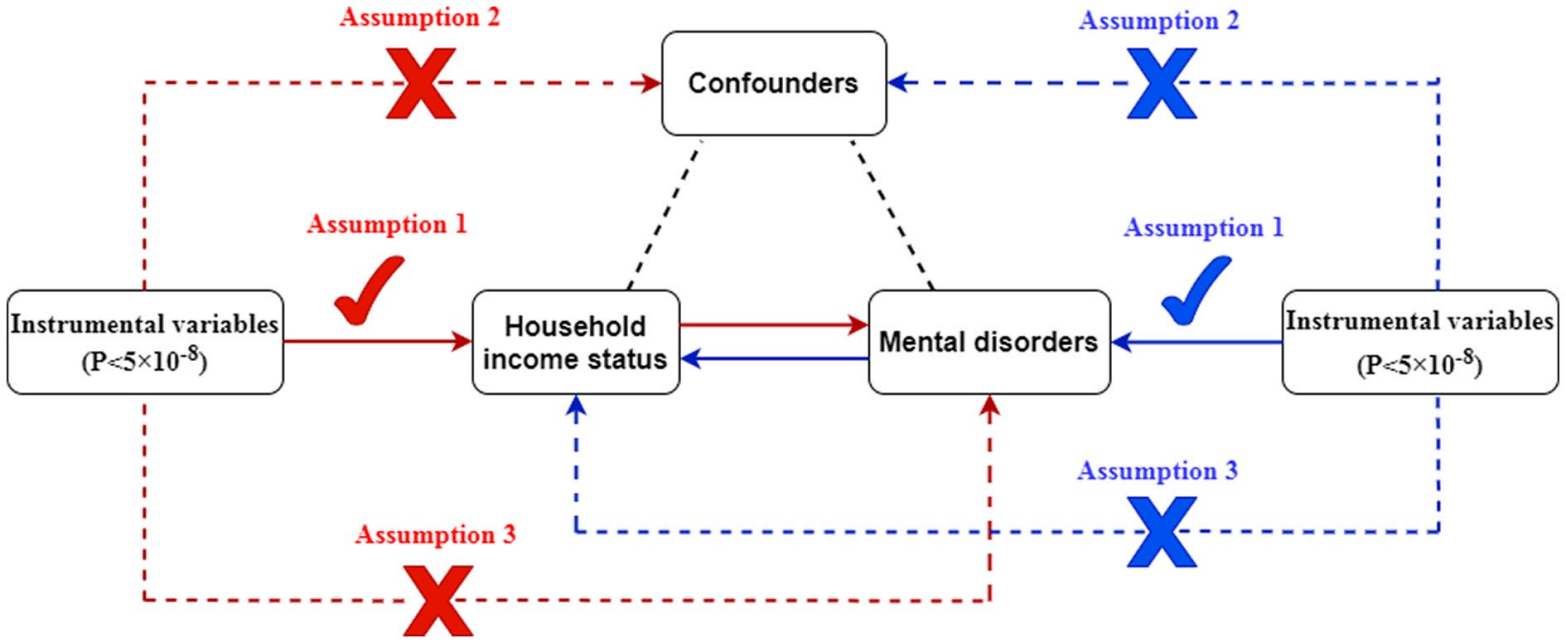
Description of the bidirectional MR study. Red represents the forward MR analyses, with household income status as exposure and mental disorders as outcomes. Blue represents the reverse MR analyses, with mental disorders as exposures and household income status as outcome.

We conducted 14 separate MR analyses designed to explore the bidirectional association between annual household income status and seven mental disorders, namely, schizophrenia, depression, anxiety disorder, panic disorder, insomnia, obsessive-compulsive disorder, and bipolar disorder. The forward MR study was performed using a random-effects inverse-variance weighting (IVW) model (16) as the primary standard and three other models [MR-Egger regression (17), weighted median (18), and maximum likelihood (19)] as supplements to evaluate the potential causal relationships between household income status as exposure factor and the seven mental disorders. The reverse MR study, applying the same standard and analysis models as the forward MR study, was performed to evaluate the potential causal relationship between the seven mental disorders as exposure factors and household income status (Figure 1).

The evidential threshold for MR analysis was defined as *p* < 0.004 (0.05/14) according to the Bonferroni correction method. *p* < 0.05 was considered significant in the sensitivity analysis. The results are reported as odds ratios (ORs) with corresponding 95% confidence intervals (CIs) and *p*-values, as well as scatter plots. The R v4.0.3 software, including the “TwoSampleMR” (20) and “MR-PRESSO” (21) packages, was used to process and visualize the study data and findings.

### 2.2 GWAS datasets information

The study was conducted using data from a large sample cohort of the European population obtained from a publicly available GWAS dataset. The genetic information used in this study was extracted from the Integrative Epidemiology Unit GWAS database^1^ (22), which is a publicly available GWAS summary database. Therefore, the requirement for ethical committee approval was waived. The GWAS summary dataset “average total household income before tax” represented the household income status of 397,751 samples originally from the UK Biobank database. The annual household income was divided into five intervals: <18,000; 18,000–30,999; 31,000–51,999; 52,000–100,000; and >100,000 pounds sterling. We followed the sample size and prioritized timeliness to make the best choices whenever possible. Detailed information on all GWAS datasets is listed in Table 1. GWAS datasets of household income and the seven mental disorders were chosen from different consortiums to decrease potential bias caused by sample overlap. In addition, to minimize racial mismatches, all GWAS datasets involved in this study predominantly included populations of European ancestry.

**Table 1:** Basic information of the GWAS datasets used in this study.

Traits	GWAS ID	Year	Population	Sample size
**Exposure factor**				**Total sample**
Household income status (23)	ukb-b-7408	2018	European	397,751
**Outcomes**				**Case/control**
Schizophrenia (24)	ieu-b-5099	2022	European	76,755/243,649
Depression (25)	finn-b-F5_DEPRESSIO	2021	European	23,424/192,220
Anxiety disorder (25)	finn-b-KRA_PSY_ANXIETY	2021	European	20,992/166,584
Panic disorder (25)	finn-b-F5_PANIC	2021	European	2,376/198,110
Insomnia (25)	finn-b-F5_INSOMNIA	2021	European	1,691/216,164
Obsessive compulsive disorder	ieu-a-1189	2017	European	26,888/7,037
Bipolar disorder (26)	ieu-b-41	2019	European	20,352/31,358

GWAS, Genome-wide association study.

### 2.3 Selection criteria for IVs

The IVs were SNPs filtered according to the afore-mentioned three pivotal assumptions of MR studies. First, the SNPs were matched using a genome-wide statistical significance threshold (*p* < 5 × 10^−8^). Second, the corresponding linkage disequilibrium was tested to confirm the presence of SNPs in the linkage disequilibrium state as well as the independence of SNPs by trimming them within a 0–10,000-kb window at a threshold of *r*^2^ < 0.001. Third, to evaluate the assumption that the IVs affect the outcomes only through the exposure factor, potential phenotypes that may have been relevant to the IVs were investigated by searching the human genotype-phenotype association database (27). Fourth, SNPs identified as IVs were further matched with those in the outcome GWAS dataset to establish genetic associations. The summary SNP-phenotype and SNP-outcome statistics were harmonized to ensure effect size alignment, and palindromic SNPs were excluded. Finally, F-statistics (>10) were used to evaluate the strength of the IVs and avoid the influence of weak instrumental bias (28).

### 2.4 Sensitivity analysis

Sensitivity analysis was performed to measure the reliability and stability of the conclusions. The sensitivity analysis consisted of (1) Cochran’s Q test (according to the IVW or MR-Egger regression models); (2) horizontal pleiotropy test using an MR-Egger intercept (29) and MR-PRESSO test (21); and (3) “leave-one-out” test (each SNP was dropped successively, and the IVW analysis was repeated to identify whether any specific SNP drove the estimate of the causal relationship).

## 3 Results

### 3.1 Forward MR study

The numbers of SNPs ultimately identified as IVs in the different outcome datasets were 42 for obsessive-compulsive disorder; 43 for schizophrenia, depression, anxiety disorder, insomnia, and panic disorder; and 44 for bipolar disorder. The F-statistic scores of all selected SNPs were >10 (obsessive-compulsive disorder: 57.43, schizophrenia: 57.64, depression: 57.77, anxiety disorder: 57.77, insomnia: 57.77, panic disorder: 57.77, and bipolar disorder: 57.49), indicating a low risk of weak instrument bias.

**Figure 2:**
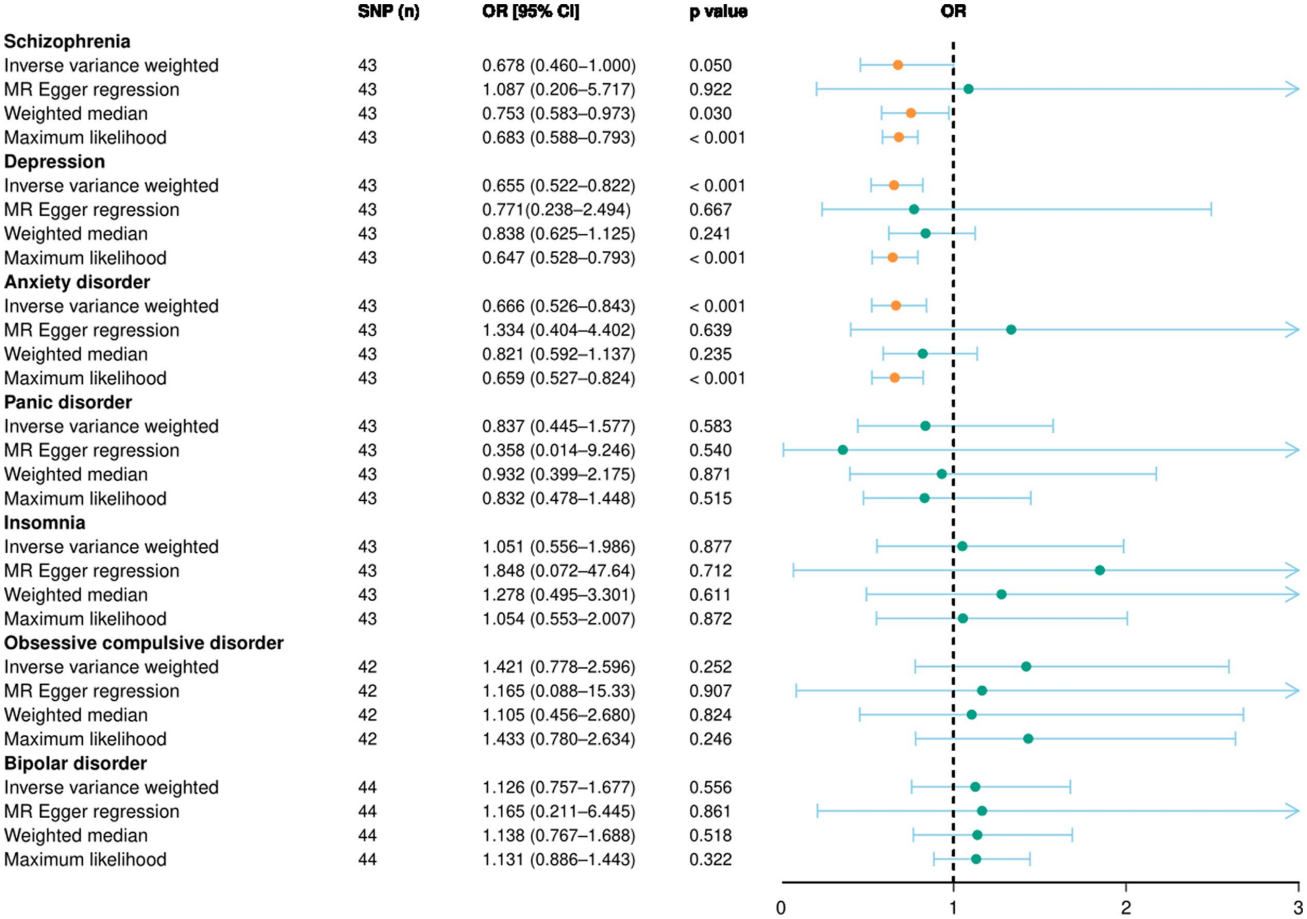
Forward MR study results illustrated by forest plot. The causal relationship between household income status and mental disorders, evaluated using an MR study. OR, odds ratio; CI, confidence interval; MR, Mendelian randomization; SNP, single nucleotide polymorphism.

Using the random-effects IVW model results as the primary standard, a higher household income tended to lower the risk of genetic liability in depression (OR: 0.655, 95% CI = 0.522–0.822, *p* < 0.001). Similarly, the result of the random-effects IVW model suggested a significant difference for anxiety disorder (OR: 0.666, 95% CI = 0.526–0.843, *p* < 0.001). These findings were supported by the maximum likelihood model. However, no significant differences were reported in the MR-Egger regression and weighted median models. In addition, no associations were observed for schizophrenia (OR: 0.678, 95% CI = 0.460–1.000, *p* = 0.05), panic disorder (OR: 0.837, 95% CI = 0.445–1.577, *p* = 0.583), insomnia (OR: 1.051, 95% CI = 0.556–1.986, *p* = 0.877), obsessive-compulsive disorder (OR: 1.421, 95% CI = 0.778–2.596, *p* = 0.252), and bipolar disorder (OR: 1.126, 95% CI = 0.757–1.677, *p* = 0.556). Detailed information is displayed in the forest plot in Figure 2 and illustrated as a scatterplot in Supplementary Figure S1.

### 3.2 Sensitivity analyses in the forward MR study

The results of Cochran’s Q test indicated heterogeneity among the IVs for schizophrenia and bipolar disorder (Table 2). However, no heterogeneity was reported for the remaining five mental disorders, especially for depression and anxiety disorder. Meanwhile, we used the random-effects IVW model to minimize the effect of heterogeneity in the MR study. No horizontal pleiotropy was detected using the MR-Egger intercept or MR-PRESSO test (Table 2). In addition, the “leave-one-out” method indicated that no specific SNP among the IVs significantly affected the overall results (Supplementary Figure S2). In general, the sensitivity analysis verified the robustness of the conclusions.

**Table 2:** Results of heterogeneity and horizontal pleiotropy tests.

Diseases	Heterogeneity test		Horizontal pleiotropy test	
	MR-Egger regression	IVW model	MR-Egger intercept	MR-PRESSO test
Schizophrenia	<0.001	<0.001	0.570	0.025
Depression	0.067	0.080	0.783	0.140
Anxiety disorder	0.247	0.233	0.252	0.116
Panic disorder	0.055	0.064	0.605	0.096
Insomnia	0.493	0.532	0.730	0.621
Obsessive compulsive disorder	0.767	0.800	0.878	0.586
Bipolar disorder	<0.001	<0.001	0.968	0.388

Sensitivity analyses consist of heterogeneity and horizontal pleiotropy tests. A *p*-value less than 0.05 was considered significant in both tests.

### 3.3 Reverse MR study and sensitivity analyses

The reverse MR study ultimately identified a total of 205, 13, 1, and 0 SNPs as IVs for schizophrenia; bipolar disorder; depression; and anxiety disorders, panic disorder, obsessive-compulsive disorder, and insomnia, respectively.

Based on the results of the random-effects IVW model and Bonferroni correction standard, the findings of the reverse MR study suggested no reverse causal relationship between the mental disorders and household income.

## 4 Discussion

Various epidemiological studies have reported a strong correlation between economic status and mental disorders at both national and individual levels (30–32). However, the establishment of a causal relationship requires further investigation. To the best of our knowledge, this study represents a pioneering effort to explore the causal impact of household income status on mental disorders using a bidirectional two-sample MR study design. In summary, the MR study findings indicated that individuals with higher household incomes tended to have reduced genetic liability for depression and anxiety disorders, as indicated by the random-effects IVW model and Bonferroni correction standard. However, no significant associations were observed for schizophrenia, panic disorder, insomnia, obsessive-compulsive disorder, or bipolar disorder. Additionally, the reverse MR analysis did not provide substantial evidence to suggest a potential causal effect of mental disorders on household income status.

Consistent with our findings, compelling evidence suggests a correlation between household income and the incidence of depression (33, 34). Few longitudinal studies have investigated the link between household income and depression risk. These investigations have diverged in research methodologies and outcome measures, yielding different findings. Notably, the Canadian National Population Health Survey’s longitudinal analysis identified an elevated likelihood of major depressive episodes among working men aged 35–74 years with low household incomes, in contrast to their peers (35). Conversely, the Stockholm Youth Cohort Study reported an increased risk of depression among adolescents from economically disadvantaged households (36). Results from the Gutenberg longitudinal general study revealed a heightened risk of developing depressive symptoms 2.5 years later in individuals with lower household net incomes who were initially devoid of baseline depression symptoms (37). In contrast to these prior investigations, our study employed MR to mitigate confounding factors. Importantly, our research benefited from a substantially larger sample size, enhancing the reliability of our conclusions.

According to data from the United States Centers for Disease Control and Prevention, 15.8% of adults in families below the federal poverty line experienced depression between 2013 and 2016. By contrast, this proportion decreased to 3.5% in families with incomes above 400% of the federal poverty line (38). Depression results from a multifactorial interplay involving genetic, biochemical, neurobiological, and psychosocial factors in various dimensions (39, 40). Therefore, the relationship between household income and depression is likely to involve interactions among multiple mechanisms. Low household income can result in heightened psychological stress, diminished social support, and other associated challenges (3, 41). Individuals may face higher burdens of chronic stress and social inequality, thereby fostering an environment conducive to elevated levels of chronic inflammatory responses, a recognized risk factor for the manifestation of depression symptoms (42–45). Economic pressure and material deprivation can also result in feelings of low mood, increased self-esteem, and diminished hope (46, 47). Prolonged stress can consistently elevate the activity of stress response systems, such as the hypothalamic-pituitary-adrenal axis (48, 49). These elevations may lead to the abnormal release of adrenergic hormones such as cortisol, thereby increasing the risk of depression (50, 51). Low income may also be associated with GABAergic and serotonergic neurotransmission (52). Depression is linked to abnormalities in the function of these neurotransmitter systems, and low-income individuals may have abnormal activity in these systems, thereby facilitating the development of depression (53, 54). Additionally, children from low-income families may experience more stress and adverse events, leading to changes in gene expression and increased susceptibility to depression (55–58). Financial constraints may cause difficulties for families to meet basic needs such as food, housing, and healthcare. Subsequently, inadequate nutrition and delayed brain development during childhood, stemming from poverty, can further amplify the likelihood of developing depression in adulthood (59). Based on our findings, strategies and interventions to prevent depression can be improved.

Studies on the relationship between household income and anxiety have yielded consistent findings (60, 61). Although previous studies have indicated a potential link between household income and anxiety, they were unable to establish any causality. Our study demonstrated a significant negative causal relationship between household income and anxiety, indicating that individuals with a higher household income exhibited a lower level of anxiety. A previous study reported that participants with an annual household income below $20,000 faced an elevated risk of developing anxiety during the 3-year follow-up compared with those earning $70,000 or more annually (3). The study also found that reduced household income was associated with an increased risk of mental disorders. Lower household income often engenders heightened financial stress, leading to apprehension concerning prospects (62). A higher household income typically provides greater access to healthcare, education, and social support systems. Sufficient access to these resources aids individuals to effectively manage and confront stressors, thereby reducing anxiety levels. Furthermore, a higher household income can enhance educational opportunities and bolster employment prospects and financial security (41, 63). Conversely, limited access to education and employment opportunities stemming from low household income could engender anxiety regarding the future. Families with low household incomes reside in disadvantaged conditions, rendering them more susceptible to environmental pollution, extreme temperatures, and challenging sleep environments (64). Moreover, women and children within impoverished households are at heightened risks of experiencing traumatic events and enduring violence perpetrated by other family members (65, 66). The cumulative impact of these factors increases their vulnerability to anxiety disorders.

Similar to other illnesses, depression and anxiety can potentially lead to economic consequences, resulting in direct or indirect declines in individual income (34). However, our study, which explored bidirectional causal relationships, did not observe unidirectional causal relationships between depression, anxiety, and household income. Specifically, our findings suggested that depression and anxiety may not significantly reduce household income. Currently, there is a lack of research investigating the impact of depression and anxiety on household income. Although depression and anxiety may directly affect individual income, their effects on household income may not necessarily manifest if other household members do not experience anxiety or depression. As households often include multiple earners or diverse income sources, the presence of other earners can mitigate the impact of depression and anxiety on household income. These findings imply that providing financial support to individuals living alone may be more beneficial than for those living with others.

The bidirectional MR study design has the substantial advantage of effectively avoiding the impact of reverse causality and reducing residual confounding factors. Nevertheless, several limitations inherent in this study should be recognized. First, the GWAS dataset used was primarily drawn from populations of European descent to avoid confounding factors due to population stratification. Consequently, the current findings may not be generalizable to other ethnic groups, and additional research is necessary to understand how these outcomes apply to diverse populations. Second, the biological functions of SNPs as IVs and how they aggravate depression and anxiety remain unclear and require further investigation. Finally, the current MR study did not include sub-group analysis for individuals at high risk of depression and anxiety. Incorporating such an analysis could have enhanced the study’s practical utility by offering a more comprehensive understanding of how household income affects diverse population subgroups. This, in turn, would have facilitated the development of more targeted policies and support measures. Therefore, the results of the present study should be interpreted with caution.

## 5 Conclusion

This study utilized extensive datasets comprising millions of individual samples and a bidirectional MR design to investigate the causal relationship between household income status and mental disorders. The results revealed that individuals from high-income households may have a decreased genetic liability for depression and anxiety. These findings underscore the importance of incorporating household income disparities into medical reimbursement policies and prioritizing efforts to improve equitable access to and availability of medical services for individuals residing in low-income households.

## Data availability statement

The original contributions presented in the study are included in the article/Supplementary material, further inquiries can be directed to the corresponding author.

## Ethics statement

The genetic information used in this study was extracted from the Integrative Epidemiology Unit GWAS database (https://gwas.mrcieu.ac.uk/), which is a publicly available GWAS summary database. Therefore, the requirement for ethical committee approval was waived.

## Author contributions

GL: Conceptualization, Writing – original draft. WL: Investigation, Methodology, Writing – original draft. XZ: Methodology, Software, Writing – review & editing. JL: Project administration, Supervision, Writing – review & editing.

## Footnotes

^1^ https://gwas.mrcieu.ac.uk/
