# Supplementary figures and images for: The higher the household income, the lower the possibility of depression and anxiety disorder: evidence from a bidirectional Mendelian randomization study
Source: Front Psychiatry. 2023 Nov 20;14:1264174. doi: 10.3389/fpsyt.2023.1264174 (PMC10694246; doi:10.3389/fpsyt.2023.1264174)

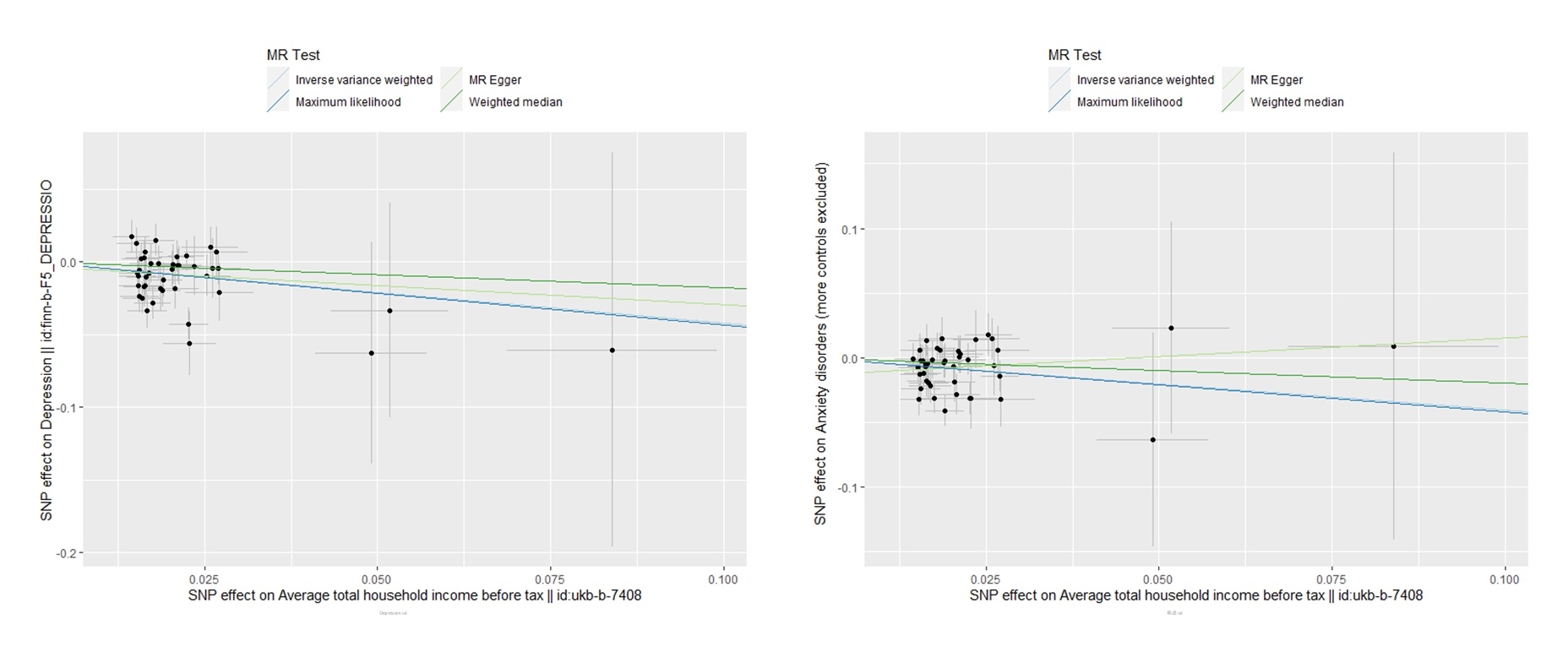

Supplement: Supplementary file 1 [file Image_1.JPEG]

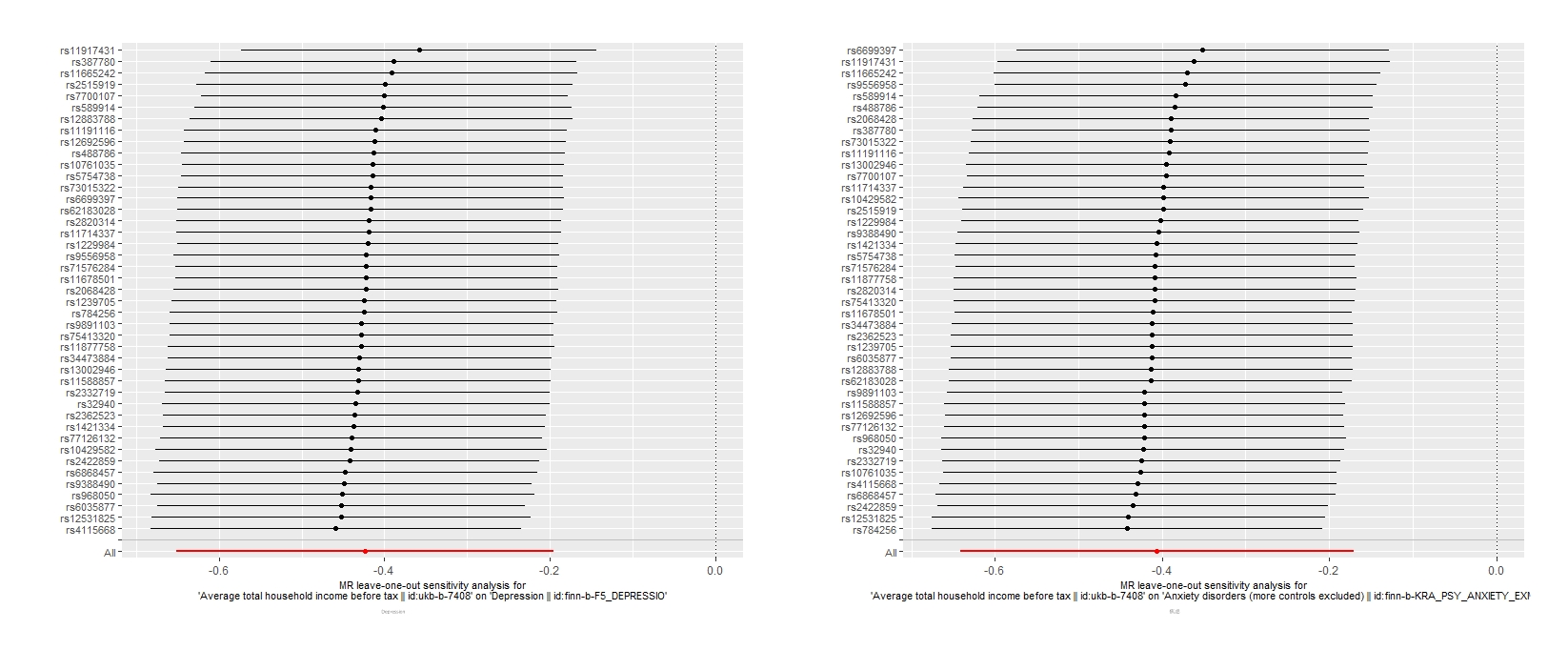

Supplement: Supplementary file 2 [file Image_2.JPEG]
